# Decreased Thigh Muscle Cross‐Sectional Area Following Anterior Cruciate Ligament Injury Depends on Concomitant Meniscal Injury and Subject Sex

**DOI:** 10.1002/jor.70247

**Published:** 2026-07-08

**Authors:** Kate French, Michael J. Toth, Niccolo Fiorentino, Pamela Vacek, Mike DeSarno, Timothy W. Tourville, Matthew Failla, Andrew Geeslin, Mathew Geeslin, Mack Gardner‐Morse, Patrick Parkinson, Bruce Beynnon

**Affiliations:** ^1^ Department of Orthopedics and Rehabilitation, McClure Musculoskeletal Research Center, Robert Larner College of Medicine University of Vermont Burlington Vermont USA; ^2^ Department of Medicine, Robert Larner College of Medicine University of Vermont Burlington Vermont USA; ^3^ Department of Mechanical Engineering, College of Engineering and Mathematical Science University of Vermont Burlington Vermont USA; ^4^ Department of Medical Biostatistics, Robert Larner College of Medicine University of Vermont Burlington Vermont USA; ^5^ Department of Rehabilitation and Movement Science, College of Nursing and Health Sciences University of Vermont Burlington Vermont USA; ^6^ Department of Radiology, Robert Larner College of Medicine University of Vermont Burlington Vermont USA

**Keywords:** anterior cruciate ligament, meniscus, muscle cross‐sectional area, muscle MRI, post‐traumatic osteoarthritis, subject sex

## Abstract

This study characterized acute changes in thigh muscle cross‐sectional area (CSA) in subjects who suffered their first severe knee trauma. Ninety‐one males and females who suffered ACL rupture with or without concomitant meniscus injury underwent bilateral mid‐thigh axial T1‐weighted, fast field echo magnetic resonance imaging early after the index trauma and prior to reconstruction. The extensor and flexor muscle groups were segmented bilaterally, and CSA measured. Within‐person injured‐to‐contralateral normal side differences in CSA (injured minus normal limb) were evaluated. ACL‐injured male and female subjects were classified into four meniscal injury groups (lateral, medial, both lateral and medial, or normal meniscus). A separate sample of healthy subjects with no history of knee injury or disease underwent the same protocol to determine the magnitude of side‐to‐side differences in thigh muscle CSA in uninjured individuals. ACL‐injured individuals had a substantially greater between‐leg differences in thigh extensor muscle CSA after injury and prior to reconstruction than healthy subjects. The between leg difference in thigh extensor muscle CSA of the injured leg was largest in males with meniscus injury involving both compartments, with males having twice as much atrophy of the extensor muscles in the injured leg compared to females in that group. The sex‐specific response of thigh muscle to severe knee trauma involving the ACL and meniscus suggests that males and females may require tailored rehabilitation programs pre‐ and post‐surgery to recover muscle size and improve muscular strength and symmetry.

## 1 Introduction

The anterior cruciate ligament (ACL) is the most common completely disrupted ligament in the knee with an injury incidence of 68.6 per 100,000, translating to more than 2 million ACL injuries occurring each year worldwide [1]. In the United States, more than 50% of knee injuries include ACL trauma, with direct and indirect costs totaling greater than $7 billion annually [2, 3]. Concomitant meniscal injury occurs in 26%–45% of patients with injury to the ACL [4]. This is a concern because severe knee trauma that involves injury to the ACL and concomitant injury to the menisci greatly increases an individual's risk of developing post‐traumatic osteoarthritis (PTOA) [5, 6, 7, 8]. As an example, ACL reconstruction (ACLR) with concomitant meniscal injury is associated with immediate changes in knee articular cartilage thickness maps, and increased rates of PTOA development over shorter time intervals than ACLR without concomitant meniscus injury [9]. This represents a considerable therapeutic concern as individuals with PTOA account for nearly 12% of all cases of symptomatic OA in the United States, they are diagnosed approximately 10 years earlier than individuals without a history of joint trauma [10, 11], and is a debilitating disease due to its early onset, predisposition to affect younger, active individuals, and the need for earlier surgical and rehabilitative interventions, all of which decrease function and quality of life.

Severe knee trauma that involves the ACL also weakens muscle, altering the magnitude and direction of intersegmental loads transmitted across the knee, and produces abnormal knee biomechanics [12]. As skeletal muscle strength is strongly dependent on muscle cross‐sectional area (CSA) [13, 14], the early loss of quadriceps strength and muscle CSA may be explained by both disuse muscle atrophy and neuromuscular changes such as arthrogenic muscle inhibition that occurs post‐injury [15, 16]. Weakness of the lower limb muscles, particularly the quadriceps, influence contact biomechanics of the articular surfaces of the knee, and creates impairments in muscle function including weakness, altered neuromuscular feedback and activation patterns, proprioceptive deficits that are commonly found in those with knee osteoarthritis (OA), and may even predispose a patient to the onset of knee OA [17, 18]. Factors contributing to the loss of muscle strength following ACL injury; however, are not clearly understood.

Skeletal muscle size is the strongest determinant of muscle strength. There are many different measurement techniques available to evaluate muscle size and composition including quantitative magnetic resonance imaging (qMRI) [19, 20], computed tomography [21], and muscle biopsy [22]. When assessing muscle CSA, T_1_ turbo spin echo (TSE) MRI is often used because it is straightforward to acquire, post‐process, and has strong image contrast between bone, fat, and muscle. MRI has the additional benefit of being able to assess intra‐ and inter‐muscular fat infiltration, which along with muscle size is associated with worsening symptoms and more degenerative changes about the knee years after the initial ACL trauma [19, 23].

Early muscle adaptations may have pathological significance, as patients with pre‐ACLR quadriceps strength deficits greater than 20% in the injured relative to the uninjured limb had larger strength deficits 2 years after ACLR than those with smaller pre‐ACLR deficits, indicating that pre‐ACLR quadriceps strength has a predictive value on post‐ACLR function [24]. One factor that can modify early skeletal muscle strength response is meniscal damage, as pain or limitations on weight bearing can cause greater muscle atrophy and weakness. While we know that knee trauma involving the ACL is associated with weakness of the quadriceps muscle, we do not understand how the severity of knee trauma (ACL vs. ACL+meniscus) affects the adaptive response of the thigh musculature, or how this adaptation is modified by other risk factors such as subject sex [25]. The objectives of our study were to characterize the acute changes in thigh muscle CSA in male and female subjects who suffered a first‐time ACL rupture, with and without concomitant meniscus injury, prior to ACLR, and to evaluate side‐to‐side symmetry of thigh muscle CSA in a separate group of normal, uninjured male and female subjects. Obtaining a better understanding of how the thigh musculature responds in the acute phase following severe knee trauma that involves the ACL and meniscus will allow development of more effective treatment paradigms that are informed by an understanding of the adaptation in skeletal muscle to the index trauma. We hypothesized that the adaptive changes of the thigh musculature following severe knee trauma differ depending on the structures involved in the injury (ACL vs*.* ACL+meniscus (lateral, medial or both medial and lateral)) and are modified by a subject's sex.

## 2 Methods

### 2.1 Participant Selection

The investigation was approved by the University of Vermont's Institutional review board, and all participants provided written informed consent prior to participation. This study contains baseline (after injury and prior to ACLR) analysis of thigh muscle CSA data acquired from an ongoing, larger prospective cohort study of the adaptive changes in thigh muscle, articular cartilage, meniscus and bone following a first‐time non‐contact ACL injury and how those changes are associated with the development of PTOA of the knee. Male and female subjects between the ages of 14 and 35 who suffered severe knee trauma involving a first‐time non‐contact ACL rupture, with and without concomitant meniscus injury, with plans to undergo arthroscopically assisted ACLR were identified immediately after diagnosis of the injury, and prior to surgery (Table 1). In total, 91 active individuals (58 females and 33 males) were recruited from high schools, colleges, sports clubs, and orthopedic and physical therapy clinics located throughout our region. The sample size was larger for the female subjects in comparison to the males, which is likely related to the two‐to‐eight fold increase in incidence of ACL injuries in females compared to males [9]. Subjects were excluded if they had any known prior knee pathology in either knee, did not fully tear their ACL, evidence of OA or apparent on MRI as assessed by a board‐certified, musculoskeletal fellowship trained radiologist, or had pre‐existing symptoms of OA in either knee.

**Table 1:** Characteristics for all subjects, females, and males. Charateristics are organized by meniscus injury group.^a^

All subjects enrolled in the study (*n* = 91)			
	Females (*n* = 58)	Males (*n* = 33)	*p*‐value
Age (years)	22.1 (6.0)	23.2 (5.7)	0.37
BMI (kg/m^2^)	25.6 (4.5)	24.6 (4.8)	0.37
Time between injury and MRI (days)	61.7 (59.3)	82.8 (75.1)	0.10
Marx total, median (min–max)	14 (0–16)	14 (1–16)	0.75
Tegner, median (min–max)	2 (0–8)	3 (1–6)	0.19
KOOS Symptoms/stiffness	61.6 (16.7)	65.4 (16.9)	0.25
KOOS Pain	72.6 (11.7)	73.5 (14.7)	0.90

Females (*n* = 58)	ACL injury and concomitant meniscus injuries (*n* = 34)			ACL injury no meniscus injury (*n* = 24)	*p*‐value
	ACL+M (*n* = 13)	ACL+L (*n* = 14)	ACL+ L+M (*n* = 7)	ACL (*n* = 24)	
Age (years)	21.8 (5.2)	22.5 (7.2)	18.7 (6.1)	22.9 (5.5)	0.29
BMI (kg/m^2^)	25.6 (4.3)	25.5 (5.0)	24.9 (3.6)	25.9 (4.8)	0.99
Time between injury and MRI: days	56.7 (39.5)	44.3 (23.6)	34.3 (21.5)	82.7 (81.0)	0.09
Marx Total, median (min–max)	16 (2–16)	16 (8–16)	16 (13–16)	13 (0–16)	0.02
Tegner, median (min–max)	2 (2–8)	3 (2–6)	2 (1–4)	2 (0–7)	0.69
KOOS symptoms/stiffness	65.4 (18.5)	57.7 (11.8)	51.5 (22.4)	64.9 (15.6)	0.32
KOOSpain	77.1 (11.5)	71.0 (11.1)	65.5 (12.7)	73.1 (11.3)	0.29

Males, (*n* = 33)	ACL injury and concomitant meniscus injuries (*n* = 21)			ACL injury no meniscus injury (*n* = 12)	*p*‐value
	ACL+M (*n* = 5)	ACL+L (*n* = 11)	ACL+L+M (*n* = 5)	ACL (*n* = 12)	
Age (years)	27.4 (4.7)	23.6 (6.9)	22.2 (4.8)	21.3 (4.9)	0.26
BMI (kg/m^2^)	24.1 (2.4)	23.7 (2.3)	25.2 (3.3)	25.5 (7.4)	0.88
Time between injury and MRI: days	77.6 (46.9)	74.3 (72.4)	99.4 (114.9)	85.9 (76.3)	0.75
Marx Total, median (min–max)	13 (9–16)	13 (9–16)	16 (13–16)	15 (1–16)	0.40
Tegner, median (min–max)	2 (2–3)	3 (2–6)	3 (1–4)	3 (1–5)	0.23
KOOS symptoms/stiffness	65.7 (11.2)	63.0 (18.2)	65.7 (14.9)	67.3 (19.8)	0.90
KOOS pain	73.3 (8.9)	75.0 (14.3)	75.0 (16.8)	71.5 (17.3)	0.97

*Note:* ACL+M indicates ACL injury with concomitant medial meniscus injury. ACL+L indicates ACL injury with concomitant lateral meniscus injury. ACL+L+M indicates ACL injury with concomitant medial and lateral meniscus injuries. ACL indicates ACL injury with no meniscus injury.

^a^ Values are provided as mean (SD [standard deviation]) unless otherwise noted. BMI, body mass index. MRI, magnetic resonance image. KOOS, knee injury and osteoarthritis outcome score. Marx Total; Marx Activity Rating Score. Tegner; Tegner Activity Score.

ACL injury was diagnosed by board‐certified orthopedic surgeons with sports medicine fellowship training and confirmed by subsequent arthroscopic visualization at the time of ACLR. Cartilage and meniscal damage, if present, were assessed at the time of arthroscopy. Demographic information, self‐reported medical history, weight, height, Tegner Activity Score (TAS), Marx Activity Rating Score (MARS), and Knee Injury and Osteoarthritis Outcome Score (KOOS) were collected from all subjects in addition to MRI prior to ACLR (Table 1).

In addition, a separate cohort of 9 healthy control subjects (5 females and 4 males) aged 18 to 26 years‐old (average 23 years) with a mean BMI of 25.25 (range: 19.9 to 30.75), that were moderately active (mean Marx activity score of 8.7) and no history of lower extremity injury or disease underwent the same bilateral thigh MRI acquisition at two time points separated by 7 days [26].

### 2.2 MRI Acquisition

Prior to positioning in the scanner, subjects were non‐weight bearing for 15 min. Measurement of thigh muscle CSA were made on the same 3 T MR scanner (Achieva, Philips Healthcare, Best, The Netherlands) with the same Philips – dStream Torso MR coil. All 91 subjects underwent bilateral mid‐femur axial 3D T1 fast field echo 3.0 T MRI (matrix: 325 × 248, slice thickness: 5 mm) scans early after the index trauma and prior to ACLR. The slice location was determined using an initial full‐thigh MR scan and locating the midpoint between the superior border of the femoral head and the inferior border of the femoral condyles allowing us to acquire bilateral scans of the thigh musculature at the same location between legs and study participants. All MR‐images were acquired by the same technologist using the same positioning (patient supine, limb unloaded with the knee in full extension and neutral thigh rotation).

### 2.3 Image Analysis

**Figure 1:**
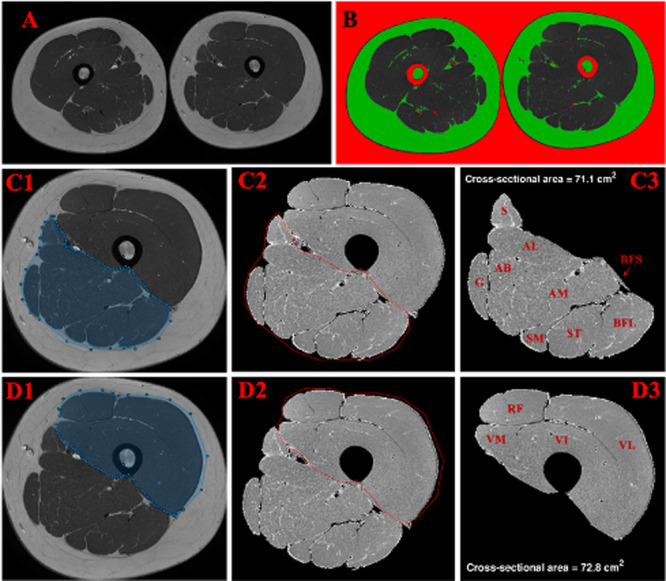
MRI thresholding and muscle segmentation using MATLAB. (A) Mid‐thigh axial T1FFE MRI acquisition. (B) MRI with thresholds applied: red = bone, green = fat, grey = muscle. Polygon ROI applied around flexor muscles (C1, C2) and extensor muscles (D1, D2). Segmentation and CSA calculation for flexor muscles (C3) and extensor muscles (D3). AB = Adductor Brevis, AL = Adductor Longus, AM = Adductor Magnus, BFL = Biceps Femoris (long head), BFS = Biceps Femoris (short head), G = Gracilis, RF = Rectus Femoris, S = Sartorius, SM = Semimembranosus, ST = Semitendinosus, VI = Vastus Intermedius, VL = Vastus Lateralis, VM = Vastus Medialis.

Bilateral extensor (rectus femoris; vastus intermedius, lateralis, medialis) and flexor (adductor brevis, longus, and magnus; biceps femoris long head and short head; gracilis; sartorius; semimembranosus; and semitendinosus) muscle groups were semiautomatically segmented, and CSA values (cm^2^) calculated using an in‐house developed MATLAB (The MathWorks, Inc., Natick, MA, USA) software program (Figure 1 and see Matlab M‐file, musc thresh ptoa.m, at https://github.com/mggardne/MRI_Muscle_Cross-Sectional_Area_PTOA). A within‐subjects design was used to evaluate, injured‐to‐contralateral normal side differences (injured minus normal limb). A single investigator blinded to the subject's sex, side of the injured limb and concomitant meniscal injury status completed all image processing and analysis.

### 2.4 Statistical Analysis

ACL‐injured male and female subjects with and without concomitant meniscus injury (*n* = 58 and *n* = 33, respectively) were classified into 4 different injury groups (ACL with lateral meniscus (ACL+L), ACL with medial meniscus (ACL+M), ACL with both medial and lateral meniscus (ACL+L+M), or ACL with a normal meniscus (ACL). Initial univariate analysies, general linear models, were conducted in order to test for significant associations between individual predictors of interest and each of the primary outcomes (injured – normal limb differences in thigh muscle CSA measures) in order to determine which covariates should be included in the final multivariate model. Analyses of covariance (ANCOVA) were used to test for significant main effects of subject sex and injury group on differences in thigh muscle CSA. Models were also run with sex‐by‐meniscus injury group (ACL, ACL+L, ACL+M, ACL+L+M) interaction terms included. In cases where the sex‐by‐meniscal interaction was found to be significant, post‐hoc pairwise comparisons were conducted, with Tukey‐Kramer correction for multiple comparisons, to test for significant effects of sex within levels of meniscal injury. BMI and KOOS Symptoms+Stiffness scores (KOOS‐SS) were included in the model as covariates. Time from injury to MRI acquisition, TAS, and MARS, were not included as covariates in the multivariate model as they were not statistically significant in univariate analyses. Statistical significance level alpha was set to 0.05. All analyses were conducted using SAS version 9.4 software (SAS Institute, Inc., Cary, NC, USA).

For the normal subjects with uninjured knees the same protocol was used to obtain the CSA measurements by a single examiner, and the data were used to establish the side‐to‐side differences in thigh muscle CSA within subjects and corresponding 95% confidence intervals (CI). These mean differences were used to determine if there were systematic differences between right and left legs and the 95% CIs were used to estimate the precision of the measurements. In an effort to provide more insight into the data from the normal, uninjured control subjecs and eliminate concerns about limb dominance effects, the differences were also calculated, and presented, as the absolute value of the right minus left thigh CSAs. We also determined the visit‐to‐visit intra‐examiner reliability of the thigh extensor and flexor muscle CSA measurements in this cohort.

## 3 Results

### 3.1 ACL‐Injured Subjects

Complete ACL tear and concomitant meniscus injury occurred in 55 of 91 participants (60%), while complete ACL without meniscus injury visible upon MRI and arthroscopic examination occurred in 36 participants (40%; Table 1). Female participants comprised 64% of the cohort (*n* = 58), and their distribution between injury groups by subject sex as well as females and males combined is presented in Table 1. Males and females did not differ by age (*p* = 0.37), had similar BMI values (*p* = 0.37), activity levels (TAS (*p* = 0.19) MARS (*p* = 0.75)), KOOS (Symptoms/stiffness (*p* = 0.25), KOOS Pain (*p* = 0.90) scores, and time between injury and MRI acquisition (*p* = 0.10) (Table 1). The sample size was larger for the female subjects in comparison to the males, which is likely related to the two‐ to eight‐fold increase in incidence of ACL injuries in active females compared to their male counterparts [27].

In ACL‐injured individuals, there was a significant main effect of meniscal injury group on injured—normal limb extensor muscle CSA difference (*p* = 0.01). In examining pairwise comparisons, there was a significantly higher mean difference in extensor muscle CSA with ACL‐injury plus concomitant meniscal trauma in both lateral and medial compartments of the knee (ACL+L+M), LSMean (SE) = 12.15 (1.57) cm^2^ than ACL trauma and a normal meniscus (ACL), LSMean (SE) = 6.13 (0.03) cm^2^ *p* = 0.01. There were no significant main effects of subject sex, or meniscal injury group on flexor muscle CSA difference or total combined extensor and flexor muscle CSA difference.

**Figure 2:**
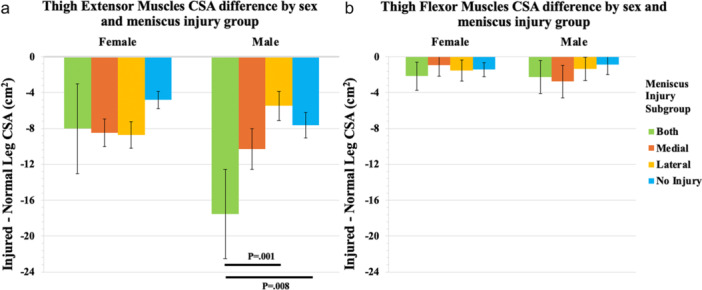
Thigh extensor muscle (left panel; 2a) and flexor muscle (right panel; 2b) cross‐sectional area (CSA) values presented by subject sex and meniscus injury group. Both = ACL+Medial and Lateral meniscus injury: Medial = ACL+Medial meniscus injury: Lateral = ACL+Lateral meniscus injury: Normal = ACL injury with no injury to the menisci. Data are presented as mean of the injured limb minus the normal limb with corresponding 95% limits of agreement.

**Figure 3:**
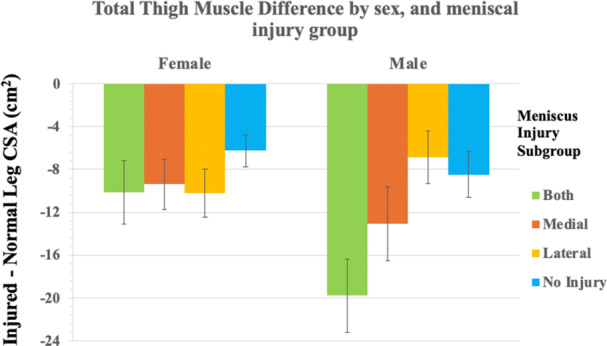
Total thigh muscle (extensors+flexors) cross‐sectional area (CSA) values presented by subject sex and meniscus injury group. Both = ACL+Medial and Lateral meniscus injury: Medial = ACL+Medial meniscus injury: Lateral = ACL+Lateral meniscus injury: Normal = ACL injury with Normal menisci. Data are presented as mean of the injured limb minus the normal limb with corresponding 95% limits of agreement.

There was a significant interaction between meniscal injury group and subject sex when evaluating the mean within‐subject extensor muscle CSA difference values (*p* = 0.01) (Figure 2a). In contrast, there were no significant interactions observed when evaluating CSA difference of the flexor muscles (*p* = 0.83) (Figure 2b) and the total combined extensor and flexor muscle groups (*p* = 0.14) (Figure 3).

When comparing males with a complete ACL‐injury plus concomitant meniscal injury in both compartments (ACL+L+M) to males with ACL‐injury plus concomitant lateral meniscal trauma (ACL+L) and to males with ACL injury and a normal meniscus (ACL), there were significant differences in mean within‐subject extensor muscle CSA (difference of least squares means (standard error) (LSMdiff) (SE) 11.71 (2.79) cm^2^, *p* = 0.001 and LSMdiff (SE) 10.12 (2.75) cm^2^, *p* = 0.01, respectively) (Figure 2a and Table 2 comparisons B and C). For both comparisons, the mean within‐subject extensor muscle CSA measurements were significantly smaller in the injured knee compared to the contralateral normal knee.

**Table 2:** Mean injured‐to‐normal limb difference (Injured limb – Normal limb) in thigh muscle cross sectional area (CSA) evaluated after injury and prior to surgery.

Subject sex	Injury group	Muscle CSA difference	Mean difference (cm^2^)	Std Dev (cm^2^)	Lower 95% CL for the Mean (cm^2^)	Upper 95% CL for the Mean (cm^2^)
Female	Both lateral and medial menisci injured	Exten CSA difference	**−9.09** ^**A**^	3.50	−5.85	−12.32
		Flex CSA Difference	−2.66	1.67	−1.11	−4.20
		Total CSA Difference	−11.73	4.74	−7.34	−16.11
	Lateral menisci injured	Exten CSA Difference	−8.90	4.43	−6.35	−11.46
		Flex CSA Difference	−1.68	3.80	−0.51	−3.88
		Total CSA Difference	−10.57	7.41	−6.29	−14.85
	Medial menisci injured	Exten CSA Difference	−7.24	6.95	−3.04	−11.44
		Flex CSA Difference	−0.82	3.78	+1.46	−3.09
		Total CSA Difference	−8.05	9.49	−2.31	−13.79
	Normal menisci	Exten CSA Difference	−4.88	3.29	−3.49	−6.27
		Flex CSA Difference	−1.27	4.02	+0.42	−2.97
		Total CSA Difference	−6.15	5.87	−3.67	−8.63
Male	Both menisci injured	Exten CSA difference	**−17.26** ^**A,B,C**^	7.69	−7.71	−26.81
		Flex CSA Difference	−2.16	3.70	+2.43	−6.76
		Total CSA Difference	−19.43	11.30	−5.39	−33.46
	Lateral menisci injured	Exten CSA Difference	**−5.69** ^**B**^	6.63	−1.23	−10.15
		Flex CSA Difference	−2.01	4.98	+2.01	−5.35
		Total CSA Difference	−7.70	9.59	+1.33	−14.14
	Medial menisci injured	Exten CSA Difference	−9.85	6.95	−1.22	−18.48
		Flex CSA Difference	−2.86	2.94	+0.80	−6.52
		Total CSA Difference	−12.71	9.09	−1.42	−24.01
	Normal menisci	Exten CSA difference	**−7.01** ^**C**^	6.05	−3.16	−10.85
		Flex CSA Difference	−0.28	5.56	+3.24	−3.82
		Total CSA Difference	−7.28	8.97	−1.58	−12.98

*Note:* All subjects had ACL injuries and data (in units of cm^2^) are presented with grouping by subject sex and the concomitant injury status of the menisci. The difference in extensor muscle CSA was significantly greater (increase loss of muscle CSA) in ACL injured males with both menisci injured compared to ACL injured females with both menisci injured (*p*‐value = 0.04 comparison A shows a difference i.e. nearly two‐fold between the sexes). Likewise, the difference in extensor muscle CSA was significantly greater in ACL injured males with both menisci injured compared to ACL injured males with a lateral meniscus injury (*p*‐value =0.001, comparison B) and ACL injured males with no meniscus injury (*p*‐value = 0.01, comparison C). In ACL‐injured females, there were no significant differences in extensor muscle CSA when comparing all four meniscal injury groups. (Exten = thigh extensors; Flex = thigh flexors, and Total = thigh extensors+thigh flexors).

In ACL‐injured females, there were no significant differences in mean within‐subject extensor muscle CSA difference values when comparing all four meniscal injury groups (Figure 2a and Table 2).

### 3.2 Healthy Subjects With Normal Limbs

There were no observable differences in CSA of the thigh extensor and flexor muscle groups between the right and left legs of the uninjured subjects with normal knees. The mean side‐to‐side differences (evaluated as right minus left thigh muscle CSA) in the extensor and flexor muscle CSA (0.28 cm^2^, CI: (−1.70 to 2.27) and 0.52 cm^2^, CI: (−1.45 to 2.49), respectively did not differ significantly from zero indicating there was no systematic difference between left and right legs (*p*‐values = 0.77 and 0.59 for extensor and flexor muscle CSA, correspondingly). In addition, to eliminate concerns about limb dominance effects, the mean absolute value of the side‐to‐side differences in the extensor and flexor muscle CSA were 1.92 cm^2^, CI: (1.05 to 2.79), and 2.03 cm^2^, CI: (0.66 to 3.40), respectively.

### 3.3 Intra‐Examiner Test‐Retest Reliability

The muscle segmentations and CSA calculations using custom, in house written MATLAB code had excellent intra‐examiner test‐retest reliability between visits with ICC values of 0.97 and 0.98 for extensor and flexor muscle groups, correspondingly.

## 4 Discussion

The main finding of this study was that the acute thigh extensor CSA reduction observed after severe knee trauma and prior to surgery differs depending on the structures involved—isolated ACL injury versus ACL with concomitant meniscal injury—and is further modified by biological sex. To our knowledge, this is the first study to characterize the acute response of thigh muscle CSA following severe knee trauma that involves ACL injury, prior to surgery, with inclusion of the modifying effects of concomitant meniscus injury, patient sex and time from injury to acquision of MRI. This has clinical significance, as decreased thigh extensor muscle CSA is associated with reduced quadriceps strength, impaired function [20, 28, 29], and altered knee joint biomechanics [12]. Compared to healthy subjects with normal knees who exhibited minimal side‐to‐side variation, ACL‐injured individuals demonstrated significant injured‐to‐normal limb asymmetry in thigh extensor muscle CSA (Figure 2a), suggesting a trauma‐specific atrophic response. Additionally, males that suffered ACL injury with concomitant injury to both menisci experienced the most pronounced muscle atrophy compared to females that suffered injury to the ACL and concomitant injury to both menisci (Table 2, comparison A). For this injury group, the underlying cause of the large difference between the sexes was not explained by the KOOS pain and symptoms/stiffness scores as these were considerably worse for the females compared to the males. It may be that the large difference between the sexes was produced by how the male musculoskeletal system responds to severe knee trauma compared to the female musculoskeletal system. Sex‐specific responses suggests that males and females may benefit from sex specific treatment programs and rehabilitation strategies pre‐ and post‐ ACLR surgery to prevent early muscle atrophy and improve muscular strength and symmetry prior to returning to pre‐injury activities.

The findings from this study suggest that it may not be appropriate to combine data from both sexes when studying the neuromuscular response of the thigh muscles to severe knee trauma that involves the ACL and menisci, as the knee extensor muscle CSA of each sex appears to undergo a different response that depends on the type of meniscus injury. This finding is supported by recent studies reporting differences in skeletal muscle between sexes have the potential to produce a differential response to knee joint trauma between females and males [30]. This is a significant clinical concern because extensor strength deficits before ACLR have been linked to poor post‐operative outcomes and lower rates of return‐to‐play at the pre‐injury level [24, 31].

In addition to assessment of thigh muscle CSA, the amount of intramuscular fat [32, 33, 34, 35, 36] should be considered as it may modulate muscle strength and plasticity [32, 33, 34, 37, 38, 39, 40]. While prior research has demonstrated associations between extensor muscle weakness, intramuscular fat of the knee extensors, and primary osteoarthritis [34, 35, 36], recent studies [41] did not find intramuscular fat to be associated with strength of the thigh muscles. In this baseline investigation, only muscle CSA was evaluated, as intramuscular fat infiltration typically reflects longer‐term maladaptive remodeling rather than immediate post‐injury changes. Prior research has shown that meaningful accumulation of intramuscular adipose tissue (IMAT) develops gradually and is more pronounced during chronic stages following ACL injury or reconstruction, rather than at the time of the initial trauma [19, 23]. Future longitudinal assessments of this cohort will evaluate IMAT and fibrotic remodeling to understand their evolution over time and their association with functional outcomes and PTOA risk.

Recent work by Owen et al. studied the effect of subject sex on quadriceps muscle fiber CSA [42]. They reported that following ACL injury, female subjects have greater between limb differences in muscle fiber CSA and between ‐limb strength deficits that were compareable to those of male subjects. In contrast, we found injured‐to‐normal leg differences in the thigh extensor muscles was largest in males with ACL and concomitant meniscus injury involving both compartments of the knee, with males having twice as much atrophy of the extensor muscles in the injured leg compared to females in that group. While the studies were similar from the perspective that male‐female comparisons were made, the differences in the finding between the studies may be explained by our approach of comparing different injury groups (ACL, ACL+L, ACL+M, ACL+L+M).

There was concern that our approach of analyzing the data as injured‐to‐normal side differences in CSA has the potential to result in bigger differences for larger individual that may have had larger muscle CSA (e.g. males vs. females). It was unclear if this contributed to our observation of greater CSA reduction in males compared to females, and consequently, we analyzed the data as ratios. Statistical analysis of the data as ratios resulted in the same findings as analysis of the injured‐to‐normal side differences (data presented in the Supplementary text).

It is important for us to mention that the thigh muscle CSA data were acquired from an ongoing, prospective cohort study of the adaptive changes in thigh muscle, articular cartilage, meniscus and bone following a first‐time non‐contact ACL injury with and without concomitant meniscus injury, and how those changes are associated with the mechanism of development of PTOA about the knee. These adaptive changes were measured with bilateral high‐resolution MRI imaging of the knee and thigh that required many different sequences that took two and one half hours to acquire. In an effort to minimize the time when subjects were in the scanner we chose to acquire a single slice at the midpoint of the femur to measue thigh muscle CSA at the same location within and between subjects instead of acquiring total muscle volume. Both thigh muscle CSA and volume are correlated with muscle strength, although it is recognized the correlation is stronger for muscle volume measurements in comparison to CSA. We chose a single slice at the midpoint of the femur as it has been shown to have the best correlation between thigh CSA and volume, mid‐femur CSA measures provide a reasonable approximation of physiological CSA of the thigh muscles, and mid‐femur CSA has a strong correlation with muscle contractile function [43]. It is also important for us to point out that it was not possible to standardize the time interval between the index injury and MRI acquisition. Consequently, we used the time interval between index injury and MRI acquisition as a covariate in the univariate analysis and found that it was not significant. The control and ACL injured subjects were of similar age and activity level. The average age of the control subjects was 23 years and this was similar to the average age of the female (22.1 years) and male (23.2 years) subjects in the current study. Similarly the control group had a similar Marx Activity Level Score, a self reported outcome measure that characterizd the subjects highest level of activity over the last year or the preinjury activity level, compared to injured subjects in the current study. In addition, this study benefits from muscle segmentation with excellent test‐retest reliability, and a healthy comparison group with normal, asymptomatic knees, no history of seeking clinical care for knee injuries or disease, and no history of lower limb injuries or disease. It is important for us to highlight that the CSA data from the healthy group was calculated as the right minus left limb differences and the absolute value of the right minus left limb to eliminate concerns associated with differences in CSA between limbs that may have been produced by limb dominance effects. Our study also has potential limitations. The concomitant meniscus injuries associated with ACL trauma were all acute, and not chronic, and as expected, primarily located in the posterior horns of the medial and lateral menisci. Most of the meniscus lesions required surgical treatment, partial resection, or a combination of the two. Meniscal repair was performed in 65.4% of the subjects, 11% underwent partial meniscectomy, and 23.6% were considered stable tears that were left untreated (these lesions did not demonstrate mechanical instability upon arthroscopic probing and did not warrant surgical repair). From this perspective a little more than three quarters of the subjects had meniscus injuries that required surgical treatment (76.4% of the cohort), while 23.6% of the cohort had stable lesions that were not treated surgically and managed according to our current standard of care. None of the subjects had isolated fraying of the meniscus as this is not common in the age range of ACL injured subjects we study in our region [44, 45]. The approach we used to create the injury groups was based on the status of the meniscus tear(s) (present or absent) and the compartment(s) in which the tear(s) was located. It would be interesting to create injury groups based on the pattern of meniscus tear (radial, circumferential, horizontal, bucke handle, flap, or complex), tear location (inner third, middle third, outer third, anterior, central, and posterior regions) and tear dimensions. This approach was not used because it would have required a far larger sample of subjects that would only be possible with a large multicenter study, and was beyond the scope of the current investigation. Our work represents the first step in beginning to understand the effect of ACL and concomitant meniscus injuries on the healing response of the thigh muscles and knee, and future work will build on this effort by establishing an approach to assess the severity of meniscus injury and define a threshold level of damage.

## 5 Significance

The decrease in knee extensor muscle CSA we observed soon after ACL and meniscus injury and prior to ACLR is likely to persist and worsen post‐reconstruction and may mark the onset of a maladaptive muscular trajectory that contributes to altered knee biomechanics in ACL‐injured individuals. This effect was most pronounced in ACL injured males with concomitant meniscal tears in medial and lateral compartments. Prior research has shown that muscle strength deficits between the injured and uninjured knees were higher for those with combined ACL+meniscus injury at 10 to 15 years follow‐up [46], and we now present evidence that this occurs soon after injury and prior to surgery. At the current point in time collection of data at the 2‐year follow‐up interval for patients who participated in this study is underway (thigh muscle CSA, thigh strength, IMAT, joint laxity, Tegner activity level, MARS, and KOOS outcomes). The longitudinal tracking of muscle size, composition, strength, and patient outcomes will be used to gain insight into the mechanism of PTOA onset and progression, and with identifying predictors of functional decline and PTOA progression following severe knee trauma that involves the ACL with and without concomitant meniscus trauma.

## Author Contributions

**Table:** 

	Substantial contribution to research design, or the acquisition, analysis and interpretation of data	Drafting the paper or revising it critically	Approval of the submitted and final versions
Kate French	X	X	X
Timothy W Tourville	X	X	X
Pamela Vacek	X	X	X
Mike DeSarno	X	X	X
Matthew Failla	X	X	X
Michael Toth	X	X	X
Niccolo Fiorentino	X	X	X
Andrew Geeslin	X	X	X
Matthew Geeslin	X	X	X
Patrick Parkinson	X	X	X
Bruce Beynnon	X	X	X

All authors have read and approved the final submitted manuscript.

## Supporting information

Supporting File

## Data Availability

The data that support the findings of this study are available on request from the corresponding author. The data are not publicly available due to privacy or ethical restrictions.
