# Supplementary material for: Decreased Thigh Muscle Cross‐Sectional Area Following Anterior Cruciate Ligament Injury Depends on Concomitant Meniscal Injury and Subject Sex
Source: J Orthop Res. 2026 Jul 8;44(7):e70247. doi: 10.1002/jor.70247 (PMC13347106; doi:10.1002/jor.70247)
Supplement: Supplementary file 1 — Supporting File [file JOR-44-0-s001.docx]

**Supplemental Text**

**Results:**

Analysis of the CSA data as ratios (normal limb CSA ratio minus the Injured limb CSA ratio / Normal limb CSA ratio) produced the same statistically significant findings as analysis of the injured – normal limb differences in thigh muscle CSA. (Table S1)

When considering the pairwise comparisons, there was a significantly higher mean CSA ratio with ACL-injury plus concomitant meniscal trauma in both lateral and medial compartments of the knee (ACL+L+M) than ACL trauma and a normal meniscus (ACL), P = 0.04. There was no significant main effect of subject sex, or meniscal injury group on flexor muscle ratio, or total combined extensor and flexor muscle CSA ratio.

There was a significant interaction between meniscal injury group and subject sex when evaluating the mean within-subject extensor muscle CSA ratio values (*P* = 0.03). In contrast, there were no significant interactions observed when evaluating CSA ratio values of the flexor muscles and the total combined extensor and flexor muscle groups.

When comparing males with a complete ACL-injury plus concomitant meniscal injury in both compartments (ACL+L+M) to males with ACL-injury plus concomitant lateral meniscal trauma (ACL+L) and to males with ACL injury and a normal meniscus (ACL), there were significant differences in mean within-subject extensor muscle CSA ratio values (P = 0.02 and P = 0.003 respectively).

In ACL-injured females, there were no significant differences in mean within-subject extensor muscle CSA ratio values when comparing all four meniscal injury groups.

Table S1: Data were collected after knee injury and prior to surgery and are presented as mean ratios of thigh muscle cross sectional area (CSA). The ratio values were calculated as the normal limb CSA minus the Injured limb CSA / Normal limb CSA. All subjects had ACL injuries and CSA ratio data (Extensor CSA ratio, Flexor CSA ratio, and Total combined Extensor and Flexor CSA ratio) are presented with grouping by subject sex and the concomitant injury status of the menisci. The ratio value for extensor muscle CSA was significantly greater (increase loss of muscle CSA) in ACL injured males with both menisci injured compared to ACL injured females with both menisci injured (p-value = 0.04 comparison A). Likewise, the ratio values of the extensor muscle CSA were significantly greater in ACL injured males with both menisci injured compared to ACL injured males with a lateral meniscus injury (p-value =.001, comparison B), and ACL injured males with no meniscus injury (p-value = .01, comparison C). In ACL-injured females, there were no significant differences in extensor muscle CSA when comparing all four injury groups. (Exten = thigh extensors; Flex = thigh flexors, and Total = thigh extensors + thigh flexors)

| **Subject**  **Sex** | **Injury Group** | **Muscle CSA Ratio**  **(Normal-Injured/Normal)*100** | **Mean Ratio**  **(%)** | **Std Dev**  **(%)** | **Lower 95% CL for the Mean (%)** | **Upper 95% CL for the Mean (%)** |
| --- | --- | --- | --- | --- | --- | --- |
| **Females** | Both Lateral and Medial Menisci  Injured | Exten CSA Ratio | - 12.67 | 4.44 | - 8.56 | - 16.77 |
|  |  | Flex CSA Ratio | - 3.38 | 1.92 | - 1.61 | - 5.15 |
|  |  | Total CSA Ratio | - 7.91 | 2.85 | - 5.28 | - 10.54 |
|  | Lateral Meniscus Injured | Exten CSA Ratio | - 13.61 | 6.79 | - 9.67 | - 17.53 |
|  |  | Flex CSA Ratio | - 2.39 | 5.39 | + 0.72 | - 5.51 |
|  |  | Total CSA Ratio | - 7.83 | 5.47 | - 4.68 | - 10.99 |
|  | Medial Meniscus Injured | Exten CSA Ratio | - 10.25 | 10.29 | - 4.02 | - 16.47 |
|  |  | Flex CSA Ratio | - 1.13 | 5.18 | + 1.99 | - 4.26 |
|  |  | Total CSA Ratio | - 5.59 | 6.77 | - 1.50 | - 9.68 |
|  | Normal Menisci | Exten CSA Ratio | - 7.78 | 5.17 | - 5.59 | - 9.97 |
|  |  | Flex CSA Ratio | - 1.55 | 6.01 | + 0.99 | - 4.09 |
|  |  | Total CSA Ratio | - 4.59 | 4.49 | - 2.69 | - 6.49 |
| **Males** | Both Lateral and Medial Menisci  Injured | Exten CSA Ratio | - 19.01 | 8.94 | - 7.91 | - 30.11 |
|  |  | Flex CSA Ratio | - 2.43 | 3.92 | + 2.43 | - 7.29 |
|  |  | Total CSA Ratio | - 10.69 | 6.25 | - 2.93 | - 18.45 |
|  | Lateral Meniscus Injured | Exten CSA Ratio | - 6.81 | 8.16 | - 1.33 | - 12.29 |
|  |  | Flex CSA Ratio | - 2.06 | 5.43 | + 1.58 | - 5.71 |
|  |  | Total CSA Ratio | - 4.41 | 5.72 | - 0.56 | - 8.26 |
|  | Medial Meniscus Injured | Exten CSA Ratio | - 11.93 | 8.05 | - 1.93 | - 21.92 |
|  |  | Flex CSA Ratio | - 3.65 | 4.11 | + 1.45 | - 8.75 |
|  |  | Total CSA Ratio | - 7.71 | 5.81 | - 0.50 | - 14.92 |
|  | Normal Menisci | Exten CSA Ratio | - 7.73 | 6.33 | - 3.71 | - 11.76 |
|  |  | Flex CSA Ratio | - 0.75 | 6.26 | + 3.23 | - 4.72 |
|  |  | Total CSA Ratio | - 4.13 | 5.03 | - 0.94 | - 7.33 |
